# Third Places for Health Promotion with Older Adults: Using the Consolidated Framework for Implementation Research to Enhance Program Implementation and Evaluation

**DOI:** 10.1007/s11524-016-0070-9

**Published:** 2016-08-25

**Authors:** Mary E. Northridge, Susan S. Kum, Bibhas Chakraborty, Ariel Port Greenblatt, Stephen E. Marshall, Hua Wang, Carol Kunzel, Sara S. Metcalf

**Affiliations:** 1 Department of Epidemiology and Health Promotion, New York University College of Dentistry, 433 First Avenue, Room 726, New York, NY 10010 USA; 2 Department of Geography, The State University of New York at Buffalo, 105 Wilkeson Quad, Buffalo, NY USA; 3 Department of Sociomedical Sciences, Columbia University Mailman School of Public Health, 722 West 168th Street, New York, NY USA; 4 Columbia University College of Dental Medicine, 630 West 168th Street, New York, NY USA; 5 Duke-National University of Singapore (Duke-NUS) Medical School, Centre for Quantitative Medicine, 20 College Road, Level 6, Academia, Singapore; 6 Department of Communication, The State University of New York at Buffalo, 359 Baldy Hall, Buffalo, NY USA

**Keywords:** Community-based programs, Health promotion, Health service delivery, Older adults, Oral health

## Abstract

This study extends the concept of third places to include community sites where older adults gather, often for meals or companionship. The Consolidated Framework for Implementation Research guided program implementation and evaluation. Depending upon health promotion program needs, the physical infrastructure of a site is important, but a supportive director (champion) can often overcome identified deficits. Senior centers may be locally classified into four types based upon eligibility requirements of residents in affiliated housing and services offered. Participants who attend these centers differ in important ways across types by most sociodemographic as well as certain health and health care characteristics.

## Introduction

Nearly three decades ago, Oldenburg introduced the concept of “great good places,” also termed “third places,” as public places on neutral ground where people may gather, enjoy the company of others, and interact.^1^^,^ 2 He contrasted the voluntary, informal, and anticipated gatherings that characterize third places to those that occur among family members in first places (home) and among employees in second places (work). In his view, beer gardens, main streets, pubs, cafés, coffeehouses, post offices, and other third places are “hangouts” that form the heart of a community.^1^^,^ 2

The potential health-promoting effects of third places have received scientific attention and preliminary study.^3^^,^ 4 Glover and Parry broadened the definition of third places by arguing that Gilda’s Club of Greater Toronto deserves this distinction, even as it is a place apart from home and *hospital* (rather than *work*) and represents a *somewhat exclusive* rather than *public* gathering place for people living with cancer.^5^ Hooper et al. also extended the concept of third places by demonstrating that creating third places from everyday environments is likely to start in *childhood*, potentially carrying on, with varying degrees of success, into adult lives.^6^ Finally, Northridge et al. posited that *online sites* might serve as third places for older adults seeking health promotion and disease management information in Harlem, New York.^7^ This may be especially important as deprived socioeconomic areas have been found to be lacking in the type of third-place amenities that might be supportive of health through encouraging contact between people.^8^

From the outset, the ElderSmile program of the Columbia University College of Dental Medicine elected to conduct its community-based oral health outreach program activities in community sites where older adults gather, i.e., third places.^9^ The foremost reason that the ElderSmile program elected to focus on creating a network of prevention centers in third places was to access a population of older adults that was not centrally interested in obtaining oral health care for painful conditions, that is, to intervene before disease is severe.^10^ Given ongoing partnership with the prevention centers over time, both older adults who received services and center directors, who value the ElderSmile program, help recruit additional participants for subsequent oral health screenings and other program activities, now including diabetes and hypertension screenings.^9^^–^ 11

While initially the ElderSmile program considered a variety of third places for prevention activities, including barber shops and other community amenities, it became clear that both social and physical infrastructure were necessary to operate the program. In thinking through the intervention and evaluation priorities for this ongoing community-based project, the Consolidated Framework for Implementation Research (CFIR) proved useful, as it provides a menu of constructs that have been associated with effective implementation and may be used in a range of applications.^12^^,^ 13 The five major domains of the CFIR are (1) the intervention characteristics, (2) the outer setting, (3) the inner setting, (4) the characteristics of the individuals involved, and (5) the process by which implementation is accomplished. Eight constructs are related to the intervention (e.g., adaptability and costs), 4 constructs are related to the outer setting (e.g., patient needs and external policy), 12 constructs are related to the inner setting (e.g., social networks and leadership engagement), 5 constructs are related to the characteristics of individuals (e.g., self-efficacy and stage of change), and 8 constructs are related to the process (e.g., planning and reflecting). See the Appendix for a full listing of the 37 CFIR constructs under 5 domains.

Guided by the CFIR, the aims of this paper are to (1) detail the rationale for why certain third places were selected for participation in the ElderSmile network, (2) introduce a locally relevant method for categorizing these third places into center types that may prove transferable or adaptable for other locales, and (3) describe the characteristics of the participants who were screened at each center type. Throughout its operation, ElderSmile has incorporated a spatial approach for addressing socioeconomic disparities in health for older adults and planning community-based health promotion activities.^14^^–^ 17 A fourth aim of this communication is thus to extend this tradition by incorporating an exploratory spatial approach to evaluate program implementation that may prove insightful for other programs that serve older adults. In the remainder of this paper, the corresponding CFIR constructs are identified in parentheses after characteristics or processes involved in the intervention. For example, the ElderSmile program (intervention) is the focus of this research.

## Methods

Throughout the course of conducting this study, all Columbia University, New York University, and University at Buffalo institutional review board and Health Insurance Portability and Accountability Act safeguards were followed.

### Selection of Third Places for the ElderSmile Network

The formation of the ElderSmile network of third places (network), also referred to as prevention centers, emerged because the foremost goal of the program, namely, to enhance the oral health of older adults in northern Manhattan, coincided with the mission of the Columbia University College of Dental Medicine, that is, to improve the oral health of its surrounding neighborhoods (external setting). In order to have the greatest community-level impact, the program founders (leaders) aimed to include a group of geographically diverse locations to ensure that the prevention centers were conveniently located within close proximity to most of the places where older adults (the individuals involved) lived. Prerequisites for site selection (the inner setting) included the following social and physical infrastructure: (1) the location needed to attract a sufficient number of older adults each day (approximately 25 or more), (2) older adults ought to congregate at the prevention center for an extended period of time (i.e., over 2 h), and (3) the site mission and director should express an interest in improving the health and quality of life of older adults. In order to conduct a successful outreach event, a prevention center also needed to have sufficient space and ideally, dedicated rooms in which to host the interactive educational slide deck presentation and perform screenings for oral disease, diabetes, and hypertension. Without sufficient space for these educational and screening activities, the outreach event flow was often hindered, and as a result, fewer participants were able to participate. Finally, the dedicated rooms needed to be well-lit and well-ventilated and provide access to electrical outlets and running water. Although few sites met all of these requirements, the ElderSmile outreach team members (formally appointed internal implementation leaders) were creative and skilled in ensuring that less than ideal physical infrastructure was nonetheless adequate to host prevention activities.

The single most important factor in determining the success of a prevention center event was the support and organization of its director (external change agent). As a result, the ElderSmile outreach team deliberately sought to develop rapport with prevention center directors toward ensuring the sustainability of the program. Time and again, our experience was that an enthusiastic and engaged center director (champion) could overcome any physical infrastructure deficits, and conversely, a disinterested and uncommitted center director could undermine even ideal physical space. Since senior centers and locations where older adults congregate were not used for the sole purpose of health screenings, other recreational activities, such as domino games and walking outside, often occurred simultaneously with outreach events. A supportive and effective director would encourage older adults to participate in ElderSmile outreach events and request that other competing activities be suspended during the educational slide deck presentation, at a minimum.

In summary, ElderSmile events held in locations with supportive leaders resulted in successful outreach activities with high participation rates, while ElderSmile events held in locations with disinterested or absentee leaders resulted in unsuccessful outreach activities with low participation rates. By way of illustration, a particularly frustrating situation occurred when the outreach team arrived at a prevention center only to discover that most of the center regulars left on a bus trip to Atlantic City, NJ. As a result, the outreach event was canceled, even though the ElderSmile outreach staff had confirmed the date multiple times with the center director.

### Locally Relevant Method for Classifying Third Places

Resources from authoritative governmental and advocacy groups for the aging were consulted in developing a locally relevant method for classifying third places for the ElderSmile network of prevention centers.^18^^,^ 19 This classification system was developed by the co-first author (S.S.K.) and verified by ElderSmile team members who also serve as co-authors. The four center types in the resulting classification system are (1) Naturally Occurring Retirement Community (NORC), (2) US Department of Housing and Urban Development Section 202 Supportive Housing for the Elderly Program (Section 202), (3) New York City Housing Authority (NYCHA), and (4) community senior or resource center (Community Center). Each of these four center types is further described in the following section.

### Measures

The individual-level data for this study were provided by self-report of participants or collected by staff and dentists of the ElderSmile program via intake interviews, clinical dental assessments, and measurement of glycosylated hemoglobin (HbA1c) and blood pressure (BP). Details of the ElderSmile clinical program and the primary-care screening enhancements are provided elsewhere.^9^^–^ 11 ^,^ 20 ^–^ 22

#### Center Type

As previously mentioned, a locally relevant method for classifying third places for older adults in urban settings was developed consisting of four center types. The first center type is *NORC*, i.e., a housing complex or neighborhood with residential dwellings that were not purposefully planned for older adults and do not restrict admission to older adults, but which house high concentrations of residents aged 60 years and older of low to moderate income, as residents have aged in place. New York State supports the following two NORC programs: (1) NORC Supportive Service Program, for housing complexes or apartment buildings that were built with government assistance, with 50 % of the units with an elderly occupant or 2500 residents aged 60 years or older, and (2) Neighborhood NORC, for a residential dwelling or group of residential dwellings in a geographically defined area with no more than 2000 people who are aged 60 years or older residing in at least 40 % of the units, made up of low-rise buildings (six stories or less), single or multi-family homes not originally built for older adults.^23^ NORCs provide support services such as health and wellness activities, socialization events, home-delivered meals, and transportation. In New York City, NORC programs are public-private partnerships with the New York City Department for the Aging, the United Hospital Fund, housing entities, community service providers, and NORC residents.^24^

The second center type is *Section 202*, i.e., a US Department of Housing and Urban Development program that offers affordable housing opportunities specifically for low- or moderate-income older adults. This program provides rent subsidies and capital advances, i.e., interest-free grants or loans, for constructing or rehabilitating housing structures. To be eligible for Section 202 housing, one person in the household must be 62 years old at the initial occupancy of the housing unit. Support services such as congregate meals, cleaning, and transportation are provided to facilitate independent living.^25^

The third center type is *NYCHA*, i.e., a public housing authority that offers affordable housing to low- and moderate-income residents in the five boroughs of New York City through the Conventional Public Housing Program or Section 8 Leased Housing Program. Housing developments and buildings exclusively for residents aged 62 years and older are available. Senior centers are located within New York City Housing Authority developments that provide recreational activities and meals for senior residents and community members in the surrounding neighborhoods. These senior centers are operated by or in partnership with New York City Housing Authority, New York City Department for the Aging, and community organizations.^26^

Finally, a senior center or resource center is a place in the community where older adults can socially interact with others and receive support services, which together comprise the fourth center type, namely, *Community Center*. Senior centers provide a range of services to adults typically aged 60 and older, notably health and wellness promotion and education. While senior centers vary in physical setting and size, populations served, services offered, and funding sources, a principal service offered by all senior centers is congregate meals—at least one meal a day, 5 days a week.^27^ Resource centers function similarly to senior centers but may not provide services or congregate meals on a regular basis.

#### Sociodemographic, Health, and Health Care Characteristics

Sociodemographic information on ElderSmile participants was obtained by questionnaire in English or Spanish, according to participant preferences. The data gathered included age, gender, race, Hispanic ethnicity, language spoken at home, place of birth, highest level of education attained, and home address. For the purposes of the analyses presented here, participants were categorized by age as 50–64, 65–74, or 75+ years. Race/ethnicity was categorized as non-Hispanic white, non-Hispanic black, Hispanic, and other. Place of birth was categorized as mainland USA, Dominican Republic, Puerto Rico, and other. The highest level of education attained was categorized as primary school, high school, or some college or more.

Health and health care information were collected by self-report or clinical assessments. Self-reported smoking status was characterized as never smoked, former smoker, or current smoker. Participants were asked if they had medical insurance (yes, no), and if so, what type (Medicaid, Medicare, or private); if they had dental insurance (yes, no), and if so, what type (Medicaid or private); and the times since their last medical and dental visits (<1, 1–3, and >3 years).

Specifically, with regard to oral health, participants assessed the status of their teeth and gums as excellent, good, fair, or poor. A faculty dentist performed a screening assessment on older adults who agreed to participate. Participants were examined for the number and condition of their teeth, and the number of teeth present was categorized as edentulous (*n* = 0), limited function (*n* = 1–19), and functional dentition (*n* = 20–28).^28^

Glycemic status (normoglycemic, pre-diabetes, or diabetes) as assessed by HbA1c was measured by a point-of-care test using capillary (finger stick) blood via a DCA Vantage Analyzer.^29^ Cut-points for measured HbA1c were as follows: normoglycemic = HbA1c < 5.6 %, pre-diabetes = HbA1c between 5.7 and 6.4 %, and diabetes = HbA1c > 6.5 %.^30^ For participants with previously diagnosed diabetes, an HbA1c of 7.0 % or higher was considered as poor glycemic control, and an HbA1c of less than 7.0 % was considered as acceptable glycemic control.

Finally, both systolic BP (SBP) and diastolic BP (DBP) were measured using an Omron blood pressure monitor.^31^ The cut-points for blood pressure status were as follows: (1) normal = SBP of 120 mmHg or less and DBP of 80 mmHg or less, (2) pre-hypertension = SBP between 120 and 139 mmHg or DBP between 80 and 89 mmHg, and (3) hypertension = SBP of 140 mmHg or more or DBP of 90 mmHg or more.^32^

### Analytic Approach

Descriptive statistics for self-reported sociodemographic, health, and health care characteristics of participants at ElderSmile outreach events held between July 2006 and October 2013 by center type were computed in *R*.^33^ Tests for the differences among participants across center types were conducted using the chi-squared test.^34^

Addresses of third places and ElderSmile participants with addresses in Manhattan and the Bronx were processed with the *Geosupport Desktop Edition*.^35^ Maps were generated with *ArcGIS Desktop*.^36^ The residential locations of participants were recorded at intake into the ElderSmile program, and the street addresses of centers were supplied by the ElderSmile program staff and verified by the co-first author (S.S.K.) via public data sources. Only participants who resided in Manhattan or the Bronx and furnished addresses without errors were georeferenced.

## Results

There were 2026 electronic records available for analysis of participants who attended ElderSmile outreach events between July 2006 and October 2013. Of these, 182 records with self-reported residential addresses that could not be georeferenced to locations in Manhattan or the Bronx, and 21 records from two ElderSmile events where the center type could not be classified with certainty, were excluded. The results presented next are based on 1823 records or 90.0 % of the original 2026 records.

### Characteristics of ElderSmile Participants Overall

The largest number and percentage of participants were seen at Community Centers (*n* = 711 or 39.0 %), followed by NYCHA, Section 202, and NORC center types (Table 1). Most of the participants were female (72.6 %) and Hispanic (57.2 %). Approximately equal percentages were aged 65–74 years (41.8 %) and greater than or equal to aged 75 years (41.0 %), even as a fair percentage was less than aged 65 years (17.3 %). While most of the participants spoke English as their primary language (53.5 %), almost as many spoke Spanish as their primary language (42.9 %). Most were born outside of the mainland USA (60.5 %) and were covered by Medicaid (51.7 %). Over a third (37.8 %) reported their highest educational attainment as primary school. Most had never smoked (58.1 %), had not visited a doctor (53.6 %) or a dentist (51.5 %) in the last year, rated their oral health as fair or poor (59.9 %), and were edentulous or had limited function dentition (64.3 %). The burden of chronic disease among participants was high, with 43.4 % self-reporting diabetes and 64.1 % with glycemic status in the pre-diabetes or diabetes range and 70.1 % self-reporting hypertension and 80.5 % with blood pressure measurements in the pre-hypertension or high range.

**TABLE 1:** Sociodemographic, health, and health care characteristics of ElderSmile participants by center type, New York, NY, 2006–2013

Characteristic	Community Center	NORC	NYCHA	Section 202	Overall	*p* Value^b^
	(*n* ^a^ = 711)	(*n* ^a^ = 125)	(*n* ^a^ = 653)	(*n* ^a^ = 334)	(*n* ^a^ = 1823)	
Gender, %						<0.01
Female	68.2	73.2	78.6	70.4	72.6	
Male	31.8	26.8	21.4	29.6	27.4	
Race/ethnicity, %						<0.01
Hispanic	53.5	43.3	65.1	54.7	57.2	
Non-Hispanic white	17.3	25.0	5.0	4.8	11.2	
Non-Hispanic black	26.0	17.5	28.0	37.6	28.2	
Other	3.2	14.2	1.9	2.9	3.5	
Age group, %						<0.01
<65 years	18.7	11.6	20.3	10.2	17.3	
65–74 years	39.2	44.6	43.3	43.3	41.8	
≥75 years	42.2	43.8	36.4	46.6	41.0	
Primary language, %						<0.01
English	54.9	56.2	48.9	58.8	53.5	
Spanish	41.2	32.2	48.9	38.8	42.9	
Other	3.9	11.6	2.2	2.5	3.6	
Place of birth, %						<0.01
Mainland USA	40.2	37.5	35.8	45.7	39.5	
Puerto Rico	19.9	12.5	28.7	21.1	22.8	
Dominican Republic	13.8	18.3	22.2	21.5	18.5	
Other	26.1	31.7	13.3	11.7	19.2	
Medicaid coverage, %						<0.01
Yes	48.8	25.2	53.4	64.1	51.7	
No	51.2	74.8	46.6	35.9	48.3	
Highest education, %						<0.01
Primary	35.5	22.5	39.6	45.2	37.8	
High school	33.3	22.5	38.9	36.9	35.2	
Some college or more	31.1	55.0	21.5	17.9	26.9	
Smoking status, %						<0.01
Never smoked	62.3	59.6	55.3	53.6	58.1	
Former smoker	29.2	35.1	29.7	32.1	30.3	
Current smoker	8.5	5.3	15.0	14.3	11.6	
Time since last visit to a doctor, %						0.09
<1 year	45.1	41.2	47.2	49.7	46.4	
1–3 years	45.9	51.3	44.1	37.5	44.1	
>3 years	9.1	7.6	8.8	12.8	9.5	
Time since last visit to a dentist, %						0.02
<1 year	48.7	59.8	48.2	44.3	48.5	
1–3 years	32.1	29.5	31.6	29.7	31.3	
>3 years	19.2	10.7	20.2	26.0	20.2	
Self-rated oral health, %						0.33
Good or better	42.4	39.4	40.0	35.5	40.1	
Fair	37.5	32.3	37.5	40.5	37.7	
Poor	20.1	28.3	22.5	24.0	22.2	
Dentition status (*n* teeth), %						<0.01
Edentulous (*n* = 0)	16.1	13.0	22.9	24.3	19.7	
Limited (*n* =1–19)	42.9	40.0	46.5	47.1	44.6	
Functional (*n* = 20–28)	41.0	47.0	30.6	28.6	35.7	
Self-reported diabetes, %						0.52
Yes	44.4	36.7	41.5	47.2	43.4	
No	55.6	63.3	58.5	52.8	56.6	
Self-reported hypertension, %						0.19
Yes	74.0	64.3	66.5	70.6	70.1	
No	26.0	35.7	33.5	29.4	29.9	
Glycemic status, %						0.82
Normal	36.2	36.6	37.9	30.8	35.9	
Pre-diabetes	39.3	36.6	34.9	40.0	37.5	
Diabetes	24.5	26.8	27.2	29.2	26.6	
Blood pressure status, %						0.29
Normal	15.5	20.9	21.6	23.2	19.4	
Pre-hypertension	39.6	46.5	36.5	35.2	38.0	
High blood pressure	44.9	32.6	41.9	41.5	42.5	

^a^Numbers may vary across characteristics because of missing values

^b^ *p* Values correspond to the testing of differences among participants across center types using the chi-squared test

### Characteristics of ElderSmile Participants by Center Type

There were significant differences by center type among ElderSmile participants by all of the sociodemographic characteristics examined (Table 1). For instance, a higher percentage of men (31.8 %) attended ElderSmile events at Community Centers than other center types, and a higher percentage of Hispanics (65.1 %) and adults aged 65 years or younger (20.3 %) attended ElderSmile events at NYCHA sites than other center types. Further, more participants at NORC sites spoke a primary language other than English or Spanish (11.6 %) or attended some college or more (55.0 %) than at other center types, while more participants at Section 202 sites were born in the mainland USA (45.7 %) than at other sites.

There were fewer significant differences by health and health care characteristics among ElderSmile participants by center type, perhaps because of the overall high burden of chronic conditions in this population. Nonetheless, more participants at Section 202 centers than other center types had Medicaid coverage (64.1 %), more participants at Community Centers than other center types never smoked (62.3 %), and more participants at NORCs had visited a dentist in the last year (59.8 %) and had functional dentition (47.0 %) than at other center types.

### Residential Locations of ElderSmile Participants by Center Type

**FIG. 1:**
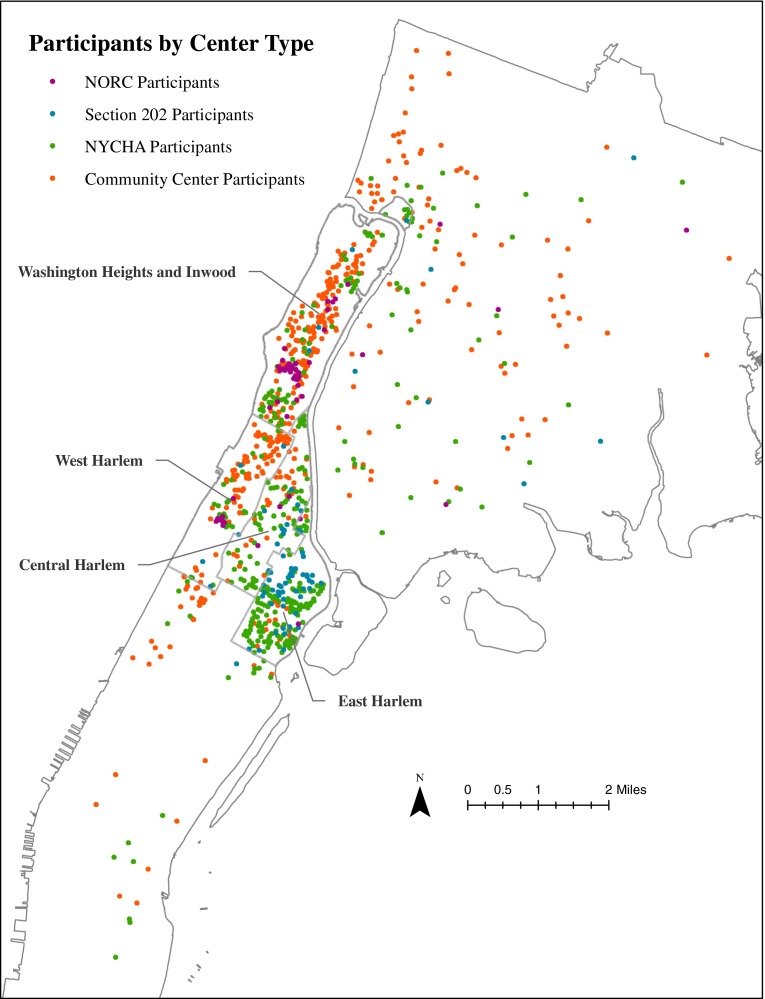
Residential locations of participants, colored to correspond to the center type where they attended an ElderSmile outreach event, New York, NY, 2006–2013.

Toward understanding where the ElderSmile participants lived and how far they travelled to attend outreach events, the residential locations of participants were mapped and colored to correspond to the center type where they attended an outreach event (Fig. 1). Most of the ElderSmile participants lived in northern Manhattan, even as they also travelled from the Bronx and locations throughout Manhattan. Clustering of participants by the center type where they attended an outreach event is more concentrated for certain center types than others; e.g., the NYCHA centers are concentrated in East Harlem.

### Residential Locations of ElderSmile Participants Who Attended Outreach Events at Community Centers

**FIG. 2:**
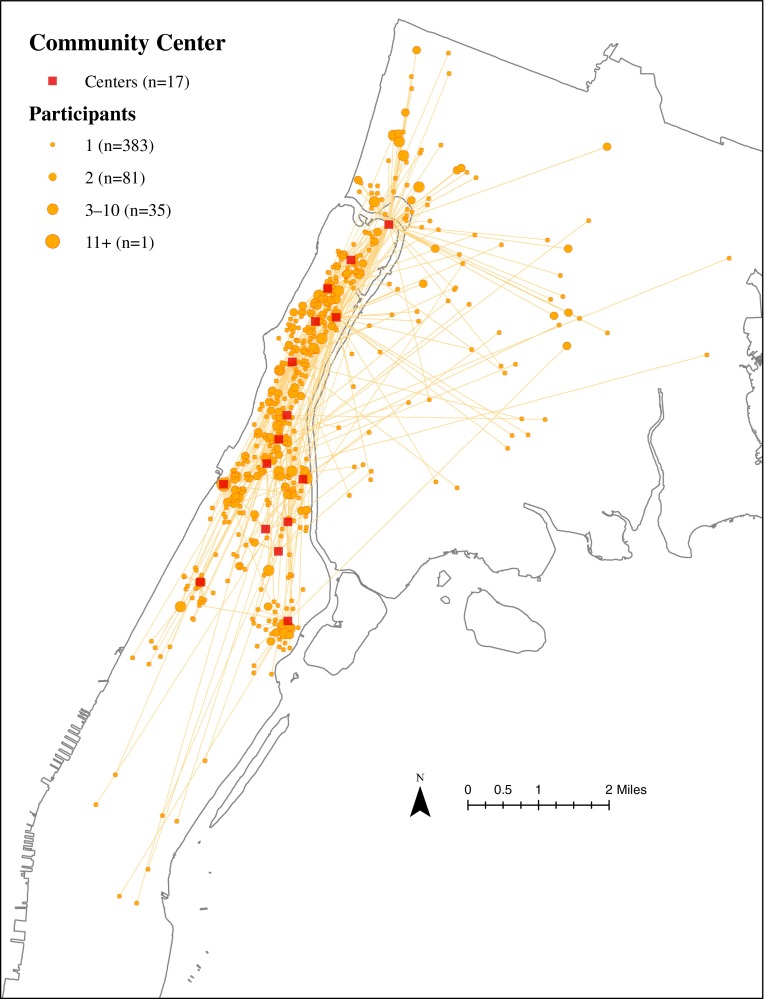
Residential locations of ElderSmile participants who attended outreach events at a community senior or resource center (*Community Center*) and the straight-line paths from their residential locations to the *Community Centers* where they attended these events, 2006–2013.

The residential locations of ElderSmile participants who attended outreach events at a Community Center and the straight-line paths from their residential locations to the Community Centers where they attended these events are depicted in Fig. 2. To indicate that there may be a number of participants living at a given residential address, weighted points were generated. These points are symbolized using graduated circles, with larger circles indicating more participants. The lines on the map are drawn from each participant’s residential location to the location of the center where s/he participated in an ElderSmile event. Community Centers drew participants from residential locations relatively far from their own locations and had the largest number of participants at a unique residential address (*n* = 383) of any center type.

### Residential Locations of ElderSmile Participants Who Attended Outreach Events at NORCs

**FIG. 3:**
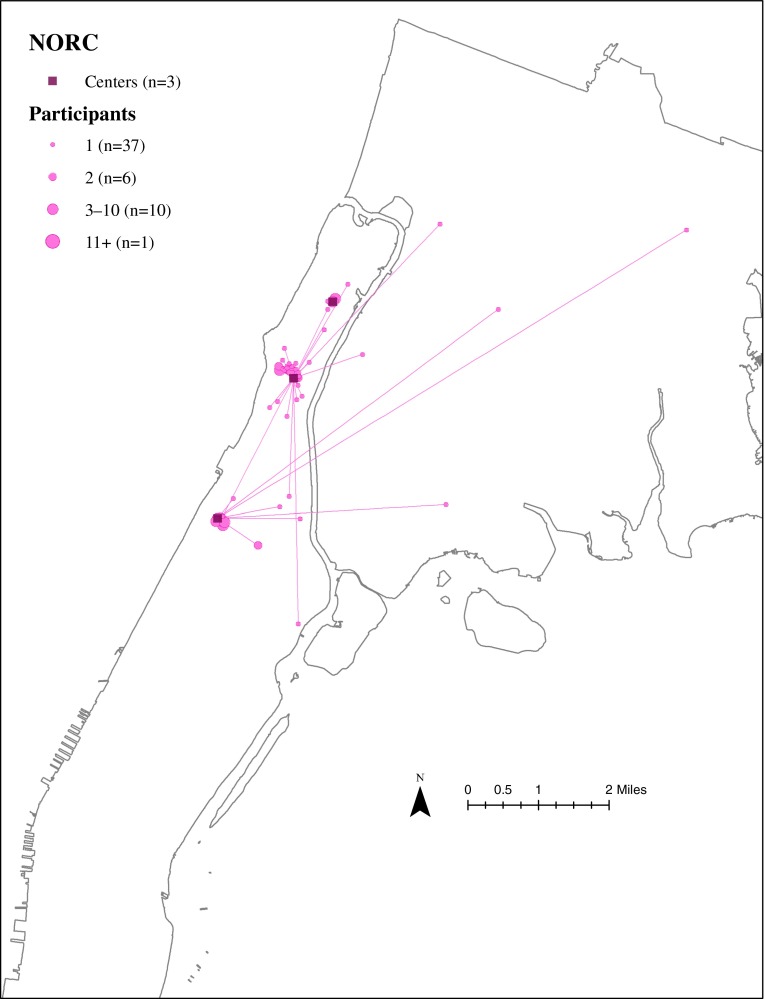
Residential locations of ElderSmile participants who attended outreach events at a Naturally Occurring Retirement Community (*NORC*) center and the straight-line paths from their residential locations to the *NORCs* where they attended these events, 2006–2013.

The residential locations of ElderSmile participants who attended outreach events at a NORC and the straight-line paths from their residential locations to the NORCs where they attended these events are depicted in Fig. 3. Note that the majority of the participants who attended an ElderSmile event at one of the three NORCs lived in close proximity to the sites. Nonetheless, the patterns reflect the operations and missions of the NORCs, the northernmost NORC primarily services residents at three affiliated housing complexes in close proximity to the site, the NORC in the center of the other two NORCs is a neighborhood NORC and services residents who live both nearby and farther away from the site, and the southernmost NORC partners with an organization that provides services in six affiliated housing complexes in a defined catchment area.

### Residential Locations of ElderSmile Participants Who Attended Outreach Events at NYCHA Centers

**FIG. 4:**
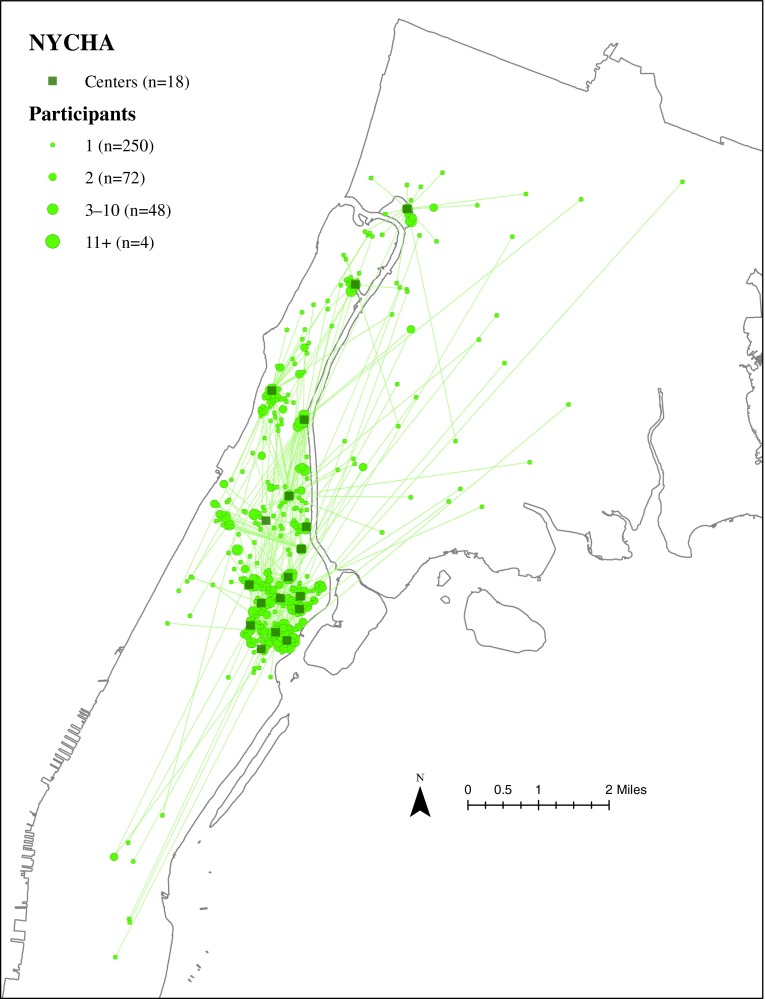
Residential locations of ElderSmile participants who attended outreach events at a New York City Housing Authority (*NYCHA*) center and the straight-line paths from their residential locations to the *NYCHA* centers where they attended these events, 2006–2013.

The residential locations of ElderSmile participants who attended outreach events at a NYCHA center and the straight-line paths from their residential locations to the NYCHA centers where they attended these events are depicted in Fig. 4. Note that there are more NYCHA centers (*n* = 18) than any other center type. NYCHA center services are open to both residents who reside at the sites as well as older adults in nearby and outlying communities.

### Residential Locations of ElderSmile Participants Who Attended Outreach Events at Section 202 Centers

**FIG. 5:**
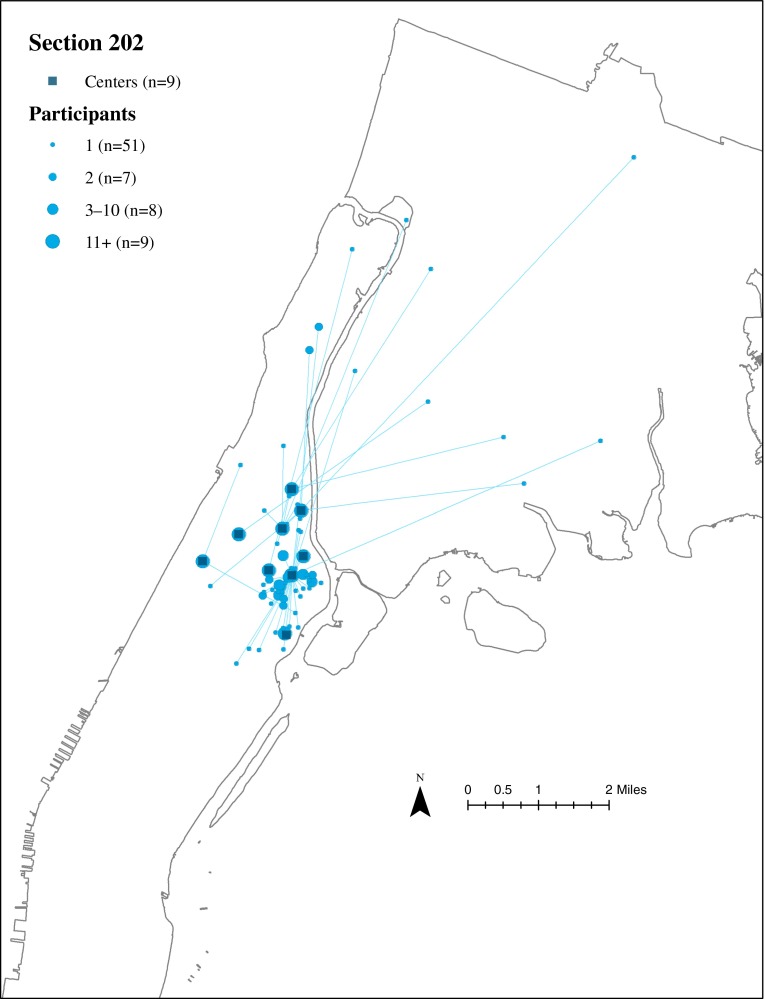
Residential locations of ElderSmile participants who attended outreach events at a US Department of Housing and Urban Development Section 202 Supportive Housing for the Elderly Program (*Section 202*) center and the straight-line paths from their residential locations to the *Section 202* centers where they attended these events, 2006–2013.

The residential locations of ElderSmile participants who attended outreach events at a Section 202 center and the straight-line paths from their residential locations to the Section 202 centers where they attended these events are depicted in Fig. 5. In contrast to the Community Centers and NYCHA centers, Section 202 centers tend to draw participants from the housing complexes with which they are affiliated.

## Discussion

A locally relevant method for categorizing third places introduced here proved useful in understanding differences among ElderSmile participants who attended outreach events by the center types they visited. Certain findings confirmed the validity of the locally relevant classification method developed as part of this research. For instance, the highest percentages of participants aged 75 years and older attended outreach events in Section 202 centers (46.6 %) and NORCs (43.8 %), and both of the housing programs with which these center types are affiliated have older age eligibility requirements. Further, the highest percentages of participants with Medicaid coverage attended outreach events in Section 202 centers (64.1 %) and NYCHA centers (53.4 %), and both of the housing programs with which these center types are affiliated have income eligibility requirements.

On the other hand, this classification system revealed unanticipated differences among participants by the center types they visited that may be useful in planning future outreach events. Note that the highest percentage of participants who had not visited a dentist for more than 3 years (26.0 %) and were edentulous (24.3 %) or had limited function dentition (47.1 %) attended outreach events at Section 202 centers. Older adults may not be aware that oral health visits are important regardless of the number of teeth they retain (reflecting).

The exploratory spatial analyses presented here add value beyond the tabulated differences among ElderSmile participants by characteristics across center types. Clearly, certain centers and types drew larger numbers of participants and had larger catchment areas than others. It is also evident that participants are not necessarily attending outreach events at the centers located closest to their residential locations. Next steps include conducting analyses using the transport network in New York City (including both train and bus routes) to estimate travel time distances for participants attending ElderSmile outreach events (planning).

### Strengths and Limitations of the Study

A major strength of this study is that these analyses were conducted in close collaboration with the ElderSmile founders, faculty dentists, and staff (implementation leaders) who had firsthand knowledge of the included centers and their directors (champions and change agents). Further, the geographers who are leaders and members of the research team possess both the expertise to conduct meaningful spatial analyses and the skills to create accessible maps to guide program implementation and evaluation (executing). Notable limitations include that we were unable to account for repeat participants at the centers and were missing essential information for 352 records that were thus not included in the analyses reported here. Further, while the classification system used in this study was locally relevant for northern Manhattan, it would need to be adapted to the context of other geographic areas, where health promotion programs are implemented for older adults. Nonetheless, there may be transferable best principles and best practices to be gleaned from our experience in implementing and evaluating the ElderSmile program.

### Interpretation and Implications of the Findings

Urban planners and public health practitioners are increasingly as focused on the social aspects of neighborhood environments as they are on the physical ones, including meeting places.^37^ In New York City, nearly one third (31.3 %) of older adults of all ages and of both genders living in NYCHA housing who responded to a telephone survey reported substantial use of senior centers, i.e., a few days a week or every day.^38^ In a representative sample of adults aged 60 years and older who attended a random selection of 56 senior centers in New York City, significant differences were found by race/ethnicity in factors related to oral health.^39^ In our study, we included center staff and caregivers accompanying older adults if they opted to participate in the ElderSmile program.

Beyond documenting attendance and need, investigators have also conducted research into implementing programs at senior centers and other community sites that address pain,^40^^,^ 41 oral health,^42^^,^ 43 stroke risk,^44^ and fall prevention.^45^^,^ 46 Third places may also be considered as anchor institutions within poor neighborhoods that foster health equity (engagement, available resources, and information access).^47^

## Conclusion

This study adds to the evidence based on evaluating place-based programs for health promotion by using the CFIR to guide program implementation and evaluation and introducing a locally relevant system for classifying center types and incorporating a spatial approach that provides insight into where participants live and how far they travel to attend outreach events at particular centers and types. We agree with Dunn that in the absence of real socioeconomic change for disadvantaged populations (external policy under outer setting), we may continue to be disappointed in the outcomes of place-based interventions.^48^ Nonetheless, action needs to occur on many levels simultaneously, and programs that reach older adults where they congregate in neighborhoods, i.e., third places, hold promise for intervening before health conditions become severe.

## Appendix

**TABLE 2:** Consolidated Framework for Implementation Research (CFIR) constructs (adapted from the CFIR Research Team^13^)

Construct		Short description
I. Intervention characteristics		
A	Intervention source	Perception of key stakeholders about whether the intervention is externally or internally developed
B	Evidence strength	Stakeholders’ perceptions of the strength of evidence that the intervention will have desired outcomes
C	Relative advantage	Stakeholders’ perception of the advantage of implementing the intervention versus an alternative solution
D	Adaptability	The degree to which an intervention can be adapted, tailored, refined, or reinvented to meet local needs
E	Trialability	The ability to test the intervention on a small scale in the organization and to be able to reverse course if warranted
F	Complexity	Perceived difficulty of implementation, reflected by duration, scope, radicalness, disruptiveness, and intricacy
G	Design packaging	Perceived excellence in how the intervention is bundled, presented, and assembled
H	Costs	Costs of the intervention and costs associated with implementation including investment, supply, and opportunity costs
II. Outer setting		
A	Patient needs	The extent to which patient needs, as well as barriers and facilitators, are accurately known and prioritized
B	Cosmopolitanism	The degree to which an organization is networked with other external organizations
C	Peer pressure	Competitive pressure to implement an intervention, typically because key peer organizations have implemented it
D	External policy	A broad construct that includes external strategies to spread interventions, including policy and regulations
III. Inner setting		
A	Structure	The social architecture, age, maturity, and size of an organization
B	Networks	The nature and quality of webs of social networks and the nature and quality of formal and informal communications
C	Culture	Norms, values, and basic assumptions of a given organization
D	Climate	The absorptive capacity for change and shared receptivity of involved individuals to an intervention
1	Tension for change	The degree to which stakeholders perceive the current situation as intolerable or needing change
2	Compatibility	The degree of tangible fit between meaning and values attached to the intervention by involved individuals
3	Relative priority	Individuals’ shared perception of the importance of the implementation within the organization
4	Incentives	Extrinsic incentives such as goal-sharing awards, performance reviews, promotions, and raises in salary
5	Goals and feedback	The degree to which goals are communicated, acted upon, and fed back to staff and alignment of feedback with goals
6	Learning climate	A climate in which there is sufficient time and space for reflective thinking and evaluation
E	Readiness	Tangible and immediate indicators of organizational commitment to its decision to implement an intervention
1	Engagement	Commitment, involvement, and accountability of leaders and managers with the implementation
2	Available resources	The level of resources dedicated for implementation and ongoing operations, including money, training, and time
3	Information access	Ease of access to digestible information about the intervention and how to incorporate it into work tasks
IV. Characteristics of individuals		
A	Knowledge and beliefs	Individuals’ attitudes toward and value placed on the intervention as well as familiarity with facts, truths, and principles
B	Self-efficacy	Individual belief in their own capabilities to execute courses of action to achieve implementation goals
C	Stage of change	Characterization of the phase an individual is in, as she progresses toward skilled, enthusiastic, and sustained use
D	Identification	A broad construct related to how individuals perceive the organization and their degree of commitment
E	Personal attributes	A broad construct to include personal traits such as tolerance of ambiguity, intellectual ability, and learning style
V. Process		
A	Planning	The degree to which a scheme and tasks for implementing an intervention are developed in advance
B	Engaging	Attracting and involving appropriate individuals in the implementation and use of the intervention
1	Opinion leaders	Individuals in an organization who have formal or informal influence on the attitudes and beliefs of their colleagues
2	Leaders	Individuals in the organization who have been formally appointed with responsibility for implementing an intervention
3	Champions	Individuals who dedicate themselves to supporting, marketing, and driving through an implementation
4	Change agents	Individuals who are affiliated with an outside entity who formally influence intervention decisions in a desirable way
C	Executing	Carrying out or accomplishing the implementation according to plan
D	Reflecting	Quantitative and qualitative feedback about the progress of implementation accompanied with regular debriefing
